# Evaluation of the Impact of *Eustenopus villosus* on *Centaurea solstitialis* Seed Production in California

**DOI:** 10.3390/insects12070606

**Published:** 2021-07-02

**Authors:** Michael J. Pitcairn, Dale M. Woods, Donald B. Joley, Charles E. Turner

**Affiliations:** 1 Biological Control Program, CDFA, Sacramento, CA 95832, USA; dalemwoods@comcast.net (D.M.W.); dbjoley@gmail.com (D.B.J.); 2 Biological Control of Weeds Laboratory, USDA-ARS, Albany, CA 94710, USA

**Keywords:** yellow starthistle, classical weed biocontrol, predispersal seed predation, hairy weevil

## Abstract

**Simple Summary:**

The exotic thistle, *Centaurea solstitialis*, has become overly abundant in the western USA. A seed weevil, *Eustenopus villosus*, was released in the early 1990s to reduce seed production and abundance of the thistle. A first step in evaluating success of the biological control program is to evaluate the amount of seed destroyed by the weevil in its new area of infestation. At two sites for two years, we found that adult weevils killed 60–70% of young flower buds, forcing plants to regrow new buds which delayed flowering by 9 days and extended flowering by 4 weeks at season end. Flower heads varied in size and seed production increased linearly by size of the flower head. The seed weevil attacked larger flower heads more frequently than smaller flower heads but the probability of a flower head being attacked did not vary with plant size. Weevil larvae occurred in 27% to 49% of seed heads and resulted in 34% to 47% of the annual seed crop being destroyed. We recommend that another survey be performed to see if the seed weevil has increased in abundance since its introduction.

**Abstract:**

The impact of the capitulum weevil *Eustenopus villosus* on *Centaurea solstitialis* seed production was examined at two field sites in central California. The study occurred in 1993–1995 during the early phases of the biological control program on *C. solstitialis* and before the current guild of capitulum insects had become widespread. Results showed that adult feeding on early flower buds resulted in 60–70% of buds failing to develop. Regrowth delayed capitulum production by 9 days and extended production by 4 weeks at season end. Between 69% and 92% of capitula were punctured from feeding or oviposition but the occurrence of larvae in capitula ranged from 27% to 49%. Seed production in *C. solstitialis* capitula increased linearly with size. The occurrence of larvae was proportionally higher in larger capitula (>8 mm) but the probability of attack for individual capitula did not vary with plant size. Total seed loss from larval feeding ranged from 34 to 47%. It is recommended that another survey be performed to determine if the level of infestation of *E. villosus* has increased since its initial introduction.

## 1. Introduction

*Centaurea solstitialis* L. (Asteraceae: Cardueae) (yellow starthistle) is an exotic annual thistle that has invaded rangelands and roadsides in several western states. It is native to southern Europe and western Eurasia and was introduced primarily as a contaminant of alfalfa seed [1]. It was first recorded in California near the San Francisco Bay Area in 1869 and now occurs in 41 US states. It is most abundant in California, Idaho, Oregon, and Washington, infesting over 6.0 million hectares in the western USA [1]. In California alone, *C. solstitialis* infests over 5.8 million hectares [2]. It is toxic to horses and can cause brain lesions that may result in the animal’s death [3,4] and reduces forage quantity and quality for cattle [5]. *Centaurea solstitialis* benefits from disturbed soils but can invade undisturbed areas where dense populations displace native and other desirable vegetation in natural ecosystems [1].

*Centaurea solstitialis* reproduces only by seed. Each inflorescence consists of a group of flowers (florets) grouped into a flower head (capitulum). A single seed (cypsela) occurs at the base of each floret and together the florets are supported on a disc (receptacle). Individual plants produce from one to hundreds of capitula [6,7] but in high density infestations commonly found in California, most plants produce less than 10 capitula. The annual seed crop is highly variable and is affected by environmental conditions [8,9] and pollination [10].

*Centaurea solstitialis* has been a target for classical biological control since 1969 when the gall fly, *Urophora jaculata* Rondani, was released in California. From 1969 through 1992, six insect species were introduced as biological control organisms (Table 1) [11]. All established (except *U. jaculata*) and all attack the capitulum and destroy developing seeds [9,11]. The seeds of *C. solstitialis* are relatively short-lived [12,13,14,15] and reduced seed production was part of a focused control strategy. Unlike other thistles in the Cardueae tribe, *C. solstitialis* does not have a dominant species within the guild of capitulum insects [16]. Field surveys in southern Europe found that the proportion of capitula attacked increased with the number of species of capitulum insects present at a location (local species richness) [16]. As a result, several species of capitulum insects were introduced to the USA in an attempt to attain the intensity of attack necessary to reduce annual recruitment and lower the abundance of *C. solstitialis*.

*Eustenopus villosus* (Boheman) is a univoltine weevil whose larvae feed on developing seeds within the capitula of *C. solstitialis*. It was released in California, Oregon, Washington, and Idaho in 1990 and 1991 [11,17] and later distributed around the western United States [18,19]. In California, *E. villosus* has become the most widespread and locally abundant of the six capitulum insects released as biological control organisms [19].

Several studies have examined the impact of *E. villosus* and the other capitulum insects on *C. solstitialis* in California [14,20], Idaho [21], and Washington [22,23,24]. Many of these studies [14,23,24] examined the combined impact of the guild of capitulum-feeding species, including *Chaetorellia succinea* L. (Diptera: Tephritidae), an accidentally introduced fruit fly that has become very common on yellow starthistle. Roche et al. [22] studied the impact of *E. villosus* using potted *C. solstitialis* plants in a field cage and Connett et al. [21] reported on the rate of infestation through the season but not seed destruction. Wooley et al. [24] estimated seed destruction by *E. villosus* and the other capitulum insects from a single sample at peak capitulum production. Garren and Strauss [14] and Swope and Satterthwaite [20] estimated the level of seed destruction due to *E. villosus* and the other introduced biological control organisms as one of several parameters for a plant population demographic model to assess the impact of seed loss on the weed population. However, there remains a lack of detailed information on the interaction of *E. villosus* with *C. solstitialis* and the within-season variation in the activity of the weevil.

Shortly after its initial release in 1990, we performed a field study to examine the impact of *E. villosus* on *C. solstitialis* seed production and phenology at two release sites in central California. While the plant material was processed and results recorded, the data were not analyzed. The study examined *E. villosus* in 1993–1995, a time prior to the accidental introduction of *C. succinea* and in the absence of interference from the other biological control organisms, a condition that no longer exists today. Prior to 1969, *C. solstitialis* in North America did not experience any feeding damage within its capitula [6,25]. Observations in other seed-herbivore systems show that following release of a seed predator, seed size can decline [26]. The results from this study uniquely document *C. solstitialis* and *E. villosus* early in the biological control program and provide baseline data with which to compare later observations. At the time of these observations, *E. villosus* was the only species present at one site and at the other site, the two other biological control organisms, *U. sirunaseva* and *B. orientalis*, were rare (infesting only two capitula).

## 2. Methods and Materials

### 2.1. Study Organisms

*Centaurea solstitialis* is a winter annual whose seed germinate following the initiation of winter precipitation. In California, precipitation usually occurs from November through March and *C. solstitialis* seed may germinate anytime during this period [7,13]. The plant occurs as a rosette until May when it produces a reproductive stalk that supports the capitula. Capitula production continues until the plant dies in late summer. Within a capitulum, the florets develop from the outer edge to the center over 4–5 days. *Centaurea solstitialis* is an obligate outcrosser, requiring pollen from other plants to produce viable seed [27,28]. Pollen transfer is dependent on insect pollinators (primarily *Apis mellifera*) for reproduction [10,29]. *Centaurea solstitialis* produces two seed types, pappus-bearing (PB): a light to dark brown form with a distinct pappus and non-pappus bearing (NPB): a dark brown to black form without a pappus. The non-pappus bearing seeds occur in a ring around the periphery of the receptacle whereas the pappus-bearing seeds occur in several rings in the center of the receptacle. Approximately 75% to 90% of the total seed output are pappus-bearing seeds and 10% to 25% are non-pappus bearing seeds [7,9]

*Eustenopus villosus* is a seed weevil native to the eastern Mediterranean area of Europe. Adult weevils emerge in late spring and undergo a period of host feeding. Adults of both sexes feed on young flower buds (<3 mm in diameter) [30,31,32]. This early bud feeding can be severe and many plants can lose all initial flower buds. Later, the plant produces new buds which develop into mature capitula on which the adult females feed and deposit their eggs. Eggs are deposited in late-stage immature buds by a female weevil chewing a hole through the phyllaries (bracts) and depositing an egg alongside a developing seed. The female seals the hole with fecal material. Damage from the oviposition puncture and larval feeding can elicit the production of a dark jelly-like substance by the host plant [33]. This substance hardens, gluing host tissue together to become a solid hard mass which can disrupt development of seeds in the affected portion of the receptacle. The developing larva feeds on young seeds and receptacle material, eventually producing a pupal chamber. Adults emerge in August and September and overwinter in plant debris nearby the host plant.

### 2.2. Study Sites

Two ungrazed field sites on opposite sides of the Central Valley of California with dense populations of *C. solstitialis* were selected as study sites. The ‘Nevada’ site was in a small open grassland surrounded by oak woodlands in the foothills of the Sierra Nevada Mountains near the town of Grass Valley in Nevada County (39.130° N, 121.022° W) (elevation 512 m). Daily summer high-temperatures averaged 26–31 °C with an average cumulative summer rainfall of 26 mm. This site was the location of the first release of *E. villosus* in North America in 1990. Our studies at this site occurred in 1993 and 1994.

The ‘Napa’ site was an open valley surrounded by oak woodlands northeast of the town of Napa in Napa County (38.403° N, 122.265° W) (elevation 427 m). Daily summer high-temperatures averaged 27–29 °C with an average cumulative summer rainfall of only 9 mm. Release of *E. villosus* occurred at this site in 1991 and our studies occurred in 1994 and 1995. Both sites remained ungrazed during the entire monitoring period and no other biological control agents were intentionally released at the sites during these field evaluations.

### 2.3. Phenology of Capitula Production and Seed Predation

Four parallel 20 m transects were established at each site across a dense area of infestation. Metal rods (*n* = 40) were placed along each transect in a stratified random method (each rod was randomly located within each half meter) for a combined total of 160 rods per site. The nearest flowering plant to each rod was selected as a study plant. Pielou [34] pointed out that selecting plants closest to random points is not truly random. However, we decided to use transects to remove observer bias in selecting plants and to minimize trampling and site disturbance. Sites were visited weekly during capitula production and for those capitula whose post-pollination florets began to degrade, a small cotton bag was tied to enclose the capitulum and confine developing seeds and any emerging insects. All capitula on all sampled plants were enclosed in bags. To increase sample size in the early and late season sample dates, additional capitula on nearby plants were bagged to increase the sample size to 100 capitula per sample date. During 1995 at the Napa site, a second site 1 km east of the original study area and where *E. villosus* had not been detected was established as a weevil-free control site (38.404° N, 122.254° W). A set of four parallel transects with 160 metal rods were placed in a dense patch of *C. solstitialis* and plants sampled as above. Extensive natural spread of the weevils precluded establishing an appropriate weevil-free study site nearby the Nevada site.

For all sites, each study plant was harvested upon death (August to October), brought to the laboratory and each capitulum on every plant was examined. External diameters of the capitula were measured and recorded in 1-mm size classes. Individual capitula were dissected under a microscope for evidence of oviposition and larval feeding damage. The number of oviposition and feeding punctures were counted and all dead *E. villosus* inside capitula were identified as larva, pupa, or adult. All seed were identified as pappus and non-pappus seed, weighed, and germinated in an environmental chamber at 20 °C on wet blotter paper with an 8/16 h light/dark cycle to determine viability. Seeds not germinating by seven days were cut open and those filled with healthy embryo tissue were considered viable. Seed output was estimated as the sum of germinated seed and ungerminated viable seed.

All non-maturing buds were examined for feeding damage. We used the terminology of Maddox [6] who described the development of *C. solstitialis* capitula in 10 identifiable stages. The immature capitula were labeled BU-1, BU-2, BU-3 and BU-4 with BU-1 as the youngest stage and the BU-4 as a mature bud with fully developed spines. All parts of individual mature capitula (without seeds) and other plant parts were oven dried at 60 °C and weighed separately. A total of 2453 capitula were bagged and evaluated during the two-year study (1993–1994) at the Nevada site and 1540 capitula were evaluated during the two years (1994–1995) at the Napa site.

### 2.4. Plant Density

Plant density was measured each year at each site using a quadrat frame (25 cm × 25 cm). At every fourth rod along the four transects (*n* = 10 rods per transect, 40 rods total), the lower right corner of the frame was located 50 cm away at a right angle from the transect and all plants and capitula within the frame were counted. The exception was for the Nevada site in 1993 where the t-square plotless distance measure was used. The distance from each rod to the selected plant and the distance of its nearest neighbor as restricted by the t-square method were recorded (*n* = 160). Plant density was estimated using the t-square calculations described by Krebs [35].

### 2.5. Statistical Evaluations

#### 2.5.1. Seed Output

Comparison of proportions for PB/NPB seed, capitula with and without punctures, capitula with and without larvae, and flower buds to mature capitula among the site/year combinations was performed with two-way contingency tables using the *G*-test of independence described in Sokal and Rohlf [36]. The production of viable seed was estimated separately for capitula with and without larval feeding damage. Most capitula had oviposition or feeding punctures but many had no internal larval feeding damage. We used capitula, with or without feeding punctures, and without larval damage to estimate seed output for unattacked capitula. There was likely some seed loss due to young florets near a puncture being damaged (usually 2–3 florets) so this is a conservative estimate of seed production for unattacked capitula. Roche et al. [22] reported a seed loss of 10% from feeding and oviposition punctures.

We examined seed output by unattacked capitula in two ways. It has been reported elsewhere [10,14] that seed output in *C. solstitialis* does not vary among capitula by size. We first examined the hypothesis that seed output increased linearly with capitulum size using Model 1 linear regression with the independent variable (size class) fixed and the dependent variable (seed output) having repeated observations. Statistical calculations followed the method described in Sokal and Rohlf [36] for the case with unequal sample sizes. To test that seed output increased linearly with capitulum size, we tested the ratio of the mean square for the linear regression to the mean square of deviations from linear regression.

#### 2.5.2. Seed Loss

For those site and year combinations for which a significant linear regression was found, we developed a step function to predict viable seed output from capitulum size. This was done because there were very few observations for the largest capitula (diameters greater than 9 mm) without *E. villosus* larvae and, as a result, it was not possible to see if the trend in seed output continued to increase, was flat, or decreased with increasing size. We decided that the best estimate of seed output for the largest capitula was the average seed output for all capitula in this grouping. For capitula from 3 mm to 9 mm in diameter, the predicted seed output was based on the linear regression of the mean number of viable seed for each 1-mm size class against the mid-size value of the size class. A test of slope equality and an unplanned comparison of slopes was performed using the GT-2 method described in Sokal and Rohlf [36]. An estimate of seed output in the absence of seed predation was produced by summing actual seed counts for capitula without larval damage and the predicted seed output in attacked capitula.

For comparison, the average seed output in attacked capitula where a pupa or adult weevil was found was calculated for each capitulum size class. This estimate represents the maximum amount of damage from the presence of *E. villosus* larvae in capitula. In capitula where the larva dies immature, the amount of feeding damage will be less. 

#### 2.5.3. Risk of Attack to Capitula

The probability of a capitulum being damaged internally by feeding *E. villosus* larvae was examined in two ways. First, for each site and year combination, the proportion of capitula with weevil larvae was plotted for each size category (outside diameter in mm). For the second method, the number of capitula per plant was used as an indicator of plant size. All capitula for plants of each size were combined and the proportion of capitula with weevil larvae were regressed against plant size. For example, all capitula from plants with only one capitulum were combined and the proportion with *E. villosus* larvae was calculated. This was done for all capitula from plants with only two capitula, etc. Before statistical analysis with linear regression, the data were transformed by taking the arcsine of the square root of each proportional value.

## 3. Results

### 3.1. Plant Density and Size

The characteristics of the *C. solstitialis* populations at the study sites for 1993–1995 are summarized in Table 2. Density and the number of capitula per plant varied greatly between years at the Nevada site with plants being larger in 1993 and producing more capitula per area. At the Napa site, plant density and the number of capitula per area was similar for both years.

### 3.2. Seed Viability and Production

Seed viability was determined by germinating seed. The amount of non-viable seed in our seed counts ranged from 2.9% to 7.8% with both seed types showing similar amounts of non-viable seed (Table 3). For seed determined as viable, the germination rate was 87% or above for both seed types for all years and sites. The ratio of the two seed types (PB seed to NPB seed) varied between sites (*G*_1,3_ = 452.2, *p* < 0.001) with a higher proportion of PB seed being produced in capitula at the Nevada site. The lowest ratio occurred at the Napa site without *E. villosus* (Table 3). For all further analyses of seed production, the number of viable seed for both seed types was combined to estimate total seed output. 

The annual seed crop among sites mirrored the variation in plant size and density. At the Nevada site, 6046 seeds per square meter (84.2 seeds per plant × 71.8 plants per square meter) were produced in 1993 but 1129 seeds per square meter were produced in 1994, a drop of 81.3%. At the Napa site with *E. villosus* present, the annual seed crops were 5252 and 4886 seeds per square meter for 1994 and 1995, respectively. At the Napa site without *E. villosus*, the annual seed crop was estimated at 7475 seeds per square meter. 

### 3.3. Impact of Eustenopus Villosus

Overwintered adult *E. villosus* emerged by early July and successfully attacked most early-season flowering buds. The proportion of buds that grew into mature capitula (success rate) varied among sites and years (*G*_1,4_ = 541.0, *p* < 0.001) (Table 4. For the four sites and years with *E. villosus*, between 30 and 40% of flower buds successfully developed to mature capitula. The exception was Napa 1994 where the success rate was 64%. In contrast, almost 90% of flower buds successfully developed to mature capitula at the *E. villosus*-free site at Napa in 1995.

Most mature capitula (68.7–91.7%) were found with feeding and oviposition punctures with the highest proportion found at the Nevada site in 1993 (*G*_1,3_ = 127.8, *p* < 0.001) (Table 5). The number of punctures in attacked capitula ranged from 1.12 to 1.58 punctures per capitulum among all years and sites (Table 5). Compared to the proportions of capitula with punctures, the proportion of capitula with larval feeding damage was substantially lower (*G*_1,3_ = 50.3, *p* < 0.001), ranging from 27.2% to 49.3% among all sites and years. The amount of decrease in the proportion of capitula with larval damage relative to the proportion of capitula with feeding and oviposition punctures varied among sites and years (*G*_1,3_ = 95.9, *p* < 0.001). The decline was similar at the Nevada site for 1993 (60%) and 1994 (66%) but at the Napa site it was substantially lower (29%) in 1994 than in 1995 (57%). Other biological control insects successfully attacked only two capitula (both in 1994 at Napa) during the years of this study. The control site remained free of all capitulum insects all season.

**Figure 1:**
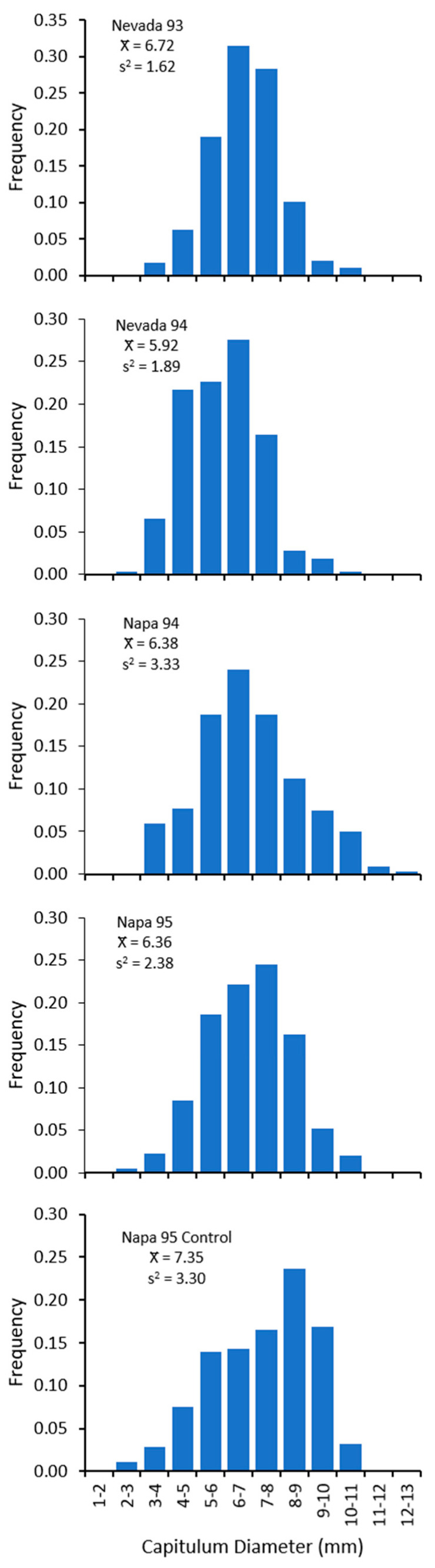
Frequency distribution of capitulum size for *Centaurea solstitialis* plants sampled from transects at two research sites during 1993 through 1995.

The size of capitula (outside diameter) produced during each season ranged from less than 3 mm to over 12 mm with most capitula between 5 and 9 mm (Figure 1). Mean capitulum size ranged from 5.92 mm to 7.35 mm with the smallest capitula occurring at the Nevada site in 1994 and the largest average occurring at the Napa site without *E. villosus* in 1995. The between-site comparison at Napa 1995 shows that the mean capitulum size of plants with *E. villosus* (6.86 mm) was smaller than capitula for plants without *E. villosus* (7.35 mm) (*n* = 667, *t* = 5.31, *p* < 0.001).

The amount of seed produced by capitula without *E. villosus* larvae increased linearly by size for all sites and years (Nevada 1993: *n* = 872, *r*^2^ = 0.22, *F*_1,6_ = 448.1, *p* < 0.001; Nevada 1994: *n* = 513, *r*^2^ = 0.16, *F*_1,6_ = 37.9, *p* < 0.001; Napa 1994: *n* = 353, *r*^2^ = 0.18, *F*_1,8_ = 20.2, *p* < 0.005; Napa 1995: *n* = 406, *r*^2^ = 0.25, *F*_1,6_ = 46.2, *p* < 0.001; Napa 1995 Control: *n* = 269, *r*^2^ = 0.31, *F*_1,6_ = 59.3, *p* < 0.001). 

The relationship between capitulum size and seed output for capitula with *E. villosus* larvae was variable. Seed production did not increase linearly with size for both years at the Nevada site (Nevada 1993: *n* = 194, *r*^2^ = 0.21, *F*_1,6_ = 4.62, *p* > 0.05; Nevada 1994: *n* = 27, *r*^2^ = 0.21, *F*_1,4_ = 1.54, *p* > 0.25) but did increase with size at the Napa site (Napa 1994: *n* = 105, *r*^2^ = 0.11, *F*_1,5_ = 12.83, *p* < 0.025; Napa 1995: *n* = 140, *r*^2^ = 0.31, *F*_1,5_ = 7.88, *p* < 0.05). 

**Figure 2:**
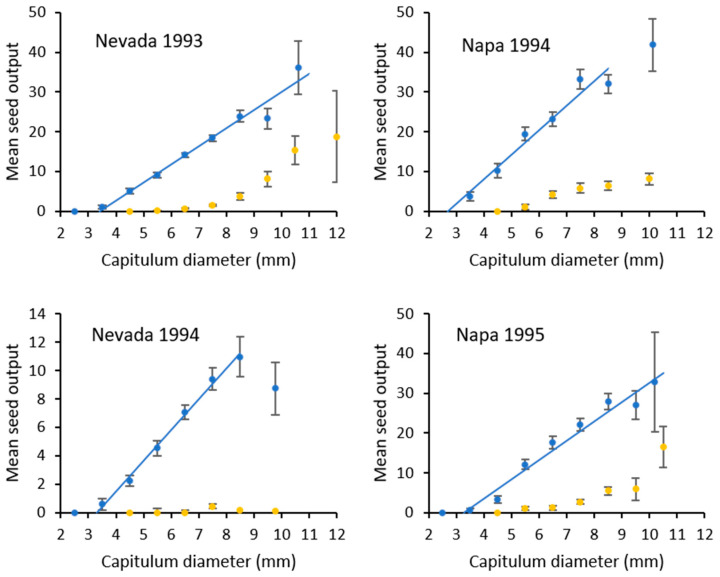
The relationship between capitulum diameter and the number of viable seed produced (mean ± SE) in attacked capitula (orange) and capitula without larval damage (blue). The regression equations are reported in Table 6.

Given the significant results above, we developed a step function based on the mean values for each size class to predict seed output for each site and year (Figure 2, Table 6). The slopes of the linear portion of these relationships were similar among years and sites except for Nevada 1994 whose capitula produced substantially fewer seed in all size classes (*F*_4,25_ = 7.286, *p* < 0.001). The mean amount of viable seed produced in capitula where *E. villosus* larvae completed development was substantially less (Figure 2). Few viable seed were produced in the smaller capitula (<7 mm) with *E. villosus* larvae but seed output increased with size except for Nevada 1994 where few viable seed were produced in capitula of all sizes with *E. villosus* larvae. The amount of viable seed produced was highest in the largest capitulum sizes (>10 mm), but these represented a small amount of the total capitula for the population (see Figure 1). 

The amounts of viable seed produced in capitula with *E. villosus* larvae shown in Figure 2 were estimated from capitula where the larva had finished feeding and had formed a pupal chamber. This represents the maximum amount of seed destroyed by a larva averaged over all infested capitula (79.0–95.0%) (Table 7). If a larva died before completing development, the amount of seed destroyed was less (76.2–85.8%).

**Figure 3:**
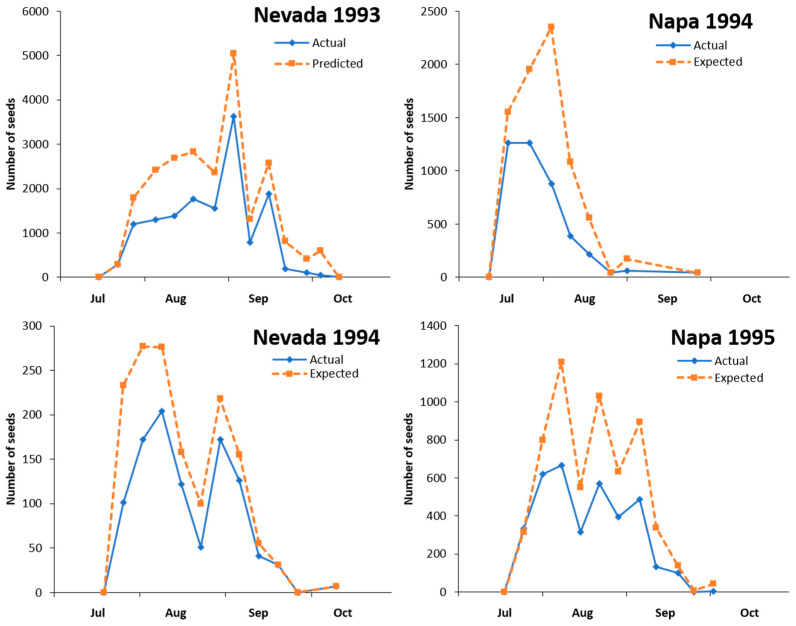
Predicted seed production in the absence of *Eustenopus villosus* larval feeding and observed seed production for two years at two field sites.

The number of observed and predicted (in the absence of *E. villosus*) viable seed produced during each season is shown in Figure 3. Seed output was variable and usually was not limited to a single peak during the season (Nevada 1994 was the exception). Seed destruction (the difference between observed and predicted values) was higher in the earlier capitula, resulting in a higher proportion of observed seed coming from smaller capitula produced later in the season. The exception was Napa 1995 where seed destruction appeared to be high throughout the season. 

The seasonal total of seed destroyed by *E. villosus* varied among the four site and year combinations (range 33.9–46.7%) (*G*_1,3_ = 106.5, *p* = 0.001) (Table 8). These values are similar to the proportion of capitula with *E. villosus* larval damage. An estimate of how efficient *E. villosus* larvae destroy seed is the ratio of proportional seed loss over the proportion of capitula with larval feeding. For most seasons, the feeding efficiency ranged from 93 to 97% (Table 8). The exception was Nevada 1994 where the amount of seed loss was higher than the proportion of capitula with *E. villosus* larvae. The higher efficiency is likely due to the proportionally higher amount of seed loss in the larger capitula and proportionally more seed coming from the smaller capitula.

### 3.4. Risk of Capitula to Attack

**Figure 4:**
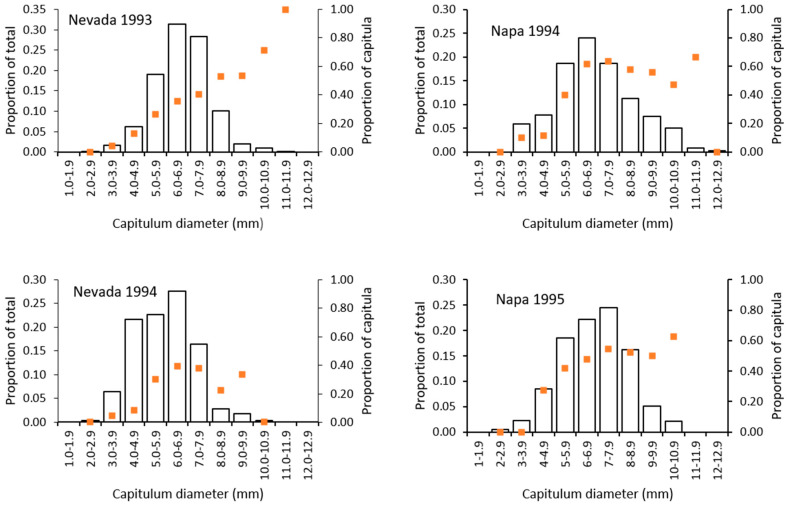
The relationship between capitulum size and probability of larval feeding by *Eustenopus villosus* for two years at two field sites. The left axis is the frequency of capitula by size (bars) and the right axis is the proportion of capitula with larval feeding damage (orange squares).

The probability of attack by capitulum size is shown in Figure 4 where the frequency distributions of capitula sizes is shown with the proportions of capitula with larval feeding. At the Nevada site in 1993, the attack rate was above 50% for capitula larger than 8.0 mm and below 50% for the smaller capitula. Similarly, the attack rate exceeded 50% for capitula larger than 6.0 mm for both years at the Napa site and was less than 50% for the smaller capitula. The exception was Nevada 1994 where the attack rate was highest in capitula between 5.0 mm and 10.0 mm but no size category exceeded a 50% attack rate.

The probability of attack for capitula growing on different plant sizes was examined by regressing the proportion of capitula with *E. villosus* larvae against the number of capitula per plant, a measure of plant size. None of the relationships were statistically significant (Nevada 1993: y = 0.034(x) + 35.12, *r*^2^ = 0.006, *F*_1,31_ = 0.174, *p* = 0.680; Nevada 1994: y = 0.264(x) + 31.17, *r*^2^ = 0.003, *F*_1,10_ = 0.035, *p* = 0.856; Napa 1994: y = 1.657(x) + 40.55, *r*^2^ = 0.266, *F*_1,9_ = 3.256, *p* = 0.105; Napa 1995: y = 0.298(x) + 40.99, *r*^2^ = 0.015, *F*_1,10_ = 0.151, *p* = 0.705), suggesting that the risk of attack for capitula was similar for large and small plants.

### 3.5. Phenology of Capitula Production

**Figure 5:**
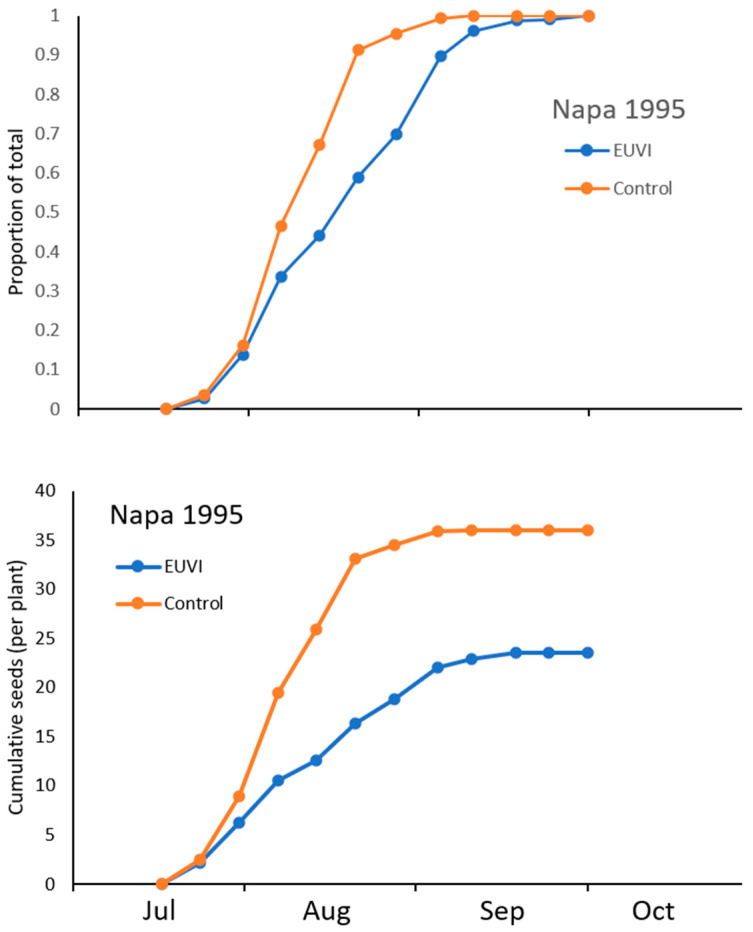
(**A**). The cumulative proportion of capitula produced by *Centaurea solstitialis* plants growing with (diamond) and without (circle) *Eustenopus villosus*. (**B**). The cumulative total of seeds per plant for the same plants in (**A**).

Feeding on the initial flower buds caused a delay in flower phenology compared to plants growing without *E. villosus*. The cumulative proportion of mature capitula produced through the season at the *E. villosus*-free site was approximately one week earlier than for plants subject to early bud damage from adult weevils (Figure 5A). At 50% of the cumulative total, the difference between the two curves is 9 days. Additionally, capitula production had essentially ended in early September at the *E. villosus*-free area but continued for four more weeks for plants at the area with *E. villosus* (Figure 5A). However, for the latter site, the amount of seed produced during the last four weeks was only 6% for the total for the season (Figure 5B), suggesting that even though some plants can grow beyond the period of activity of the weevil, the amount of seed produced late in the season is substantially less. 

## 4. Discussion

The capitulum weevil, *E. villosus*, impacts the reproduction of *C. solstitialis* in two ways. One is during the early flowering stage when adult weevils feed on young flower buds, causing the plant to regrow new buds and delay flowering. The second is the direct loss of seed from the larvae feeding inside the capitula. The attack on young flower buds resulted in 60–70% of buds failing to develop. The production of mature capitula was delayed by at least a week (at 50% production, the delay was 9 days) and was extended by four weeks at the end of the season. These late capitula contributed only 6% of the total seasonal production of seed. Spencer et al. [32] showed that early bud loss on *C. solstitialis* reduced capitulum size, which resulted in a reduction of 21% in expected seed output per plant. At the Napa site in 1995, the mean capitula diameter for plants with *E. villosus* was 7% smaller than the mean capitula diameter for plants without the weevil, suggesting that adult feeding damage of immature buds may result in smaller capitula.

As the season progresses, adult *E. villosus* shift from young flower buds to feeding on mature, unopened capitula (BU-4). In this study, the attack rates of mature capitula ranged between 69% and 92% with many capitula having multiple punctures. Despite the high puncture rates, the number of capitula with larvae was much lower, ranging between 27% and 49%. In a study comparing herbicide-resistant and -susceptible *C. solstitialis* plants, Roche et al. [22] reported 97% of capitula with punctures (same for both plant types) and 56% and 37% of capitula with larvae for the resistant and susceptible plants, respectively. The difference between puncture rates and larval infestation rates may be due to the lack of deposition of eggs. Punctures in mature capitula result from either simple feeding or oviposition [31,33] and, in a separate study, we found that 63% (range 60–67%) of punctures resulted in egg deposition (unpublished data). Another possibility is that eggs and young larvae experience high rates of mortality. Oviposition in a capitulum can sometimes cause a wound response where the plant produces a dark jelly-like substance that hardens within the capitulum [33]. This response can result in mortality of the egg and young larvae. Swope and Satterthwaite [20] reported that the generalist parasitic mite, *Pyemotes tritici*, resulted in mortality of *E. villosus* larvae of 25–38%, however, the mite was not observed during our study. 

The highest amount of seed loss was due to larval feeding within the capitulum. Seed production by *C. solstitialis* increased linearly with capitulum size and, when present, a *E. villosus* larva can destroy a substantial amount of seed. Capitulum size was variable among plants and over the flower season ranging from 2.5 to 12.5 mm outside diameter with peak abundance from 5.5 to 7.5 mm in diameter. For capitula less than 6 mm diameter, which represented approximately half of the capitula produced in a population, most or all of the seed are removed when a larva is present. Some seeds remain undamaged in the larger capitula but when averaged over all capitula, seed loss per capitulum was between 79% and 95% when the larva completed feeding and formed a pupal cell. These values are higher than the 71.4% seed loss per capitulum reported by Woodley et al. [24] and the 66.8% and 67.2% seed loss per capitulum reported by Swope and Satterthwaite [20]. The differences may be due to these studies sampling larger capitula. 

The ratio of pappus-bearing to non-pappus-bearing seed ranged from 2.09 to 3.78 PB/NPB for the four site-year combinations in our study and these ratios are much lower than reported elsewhere. Roche et al. [22] reported 4.1 to 7.4 PB/NPB for plants with *E. villosus* present and 4.5 to 6.5 PB/NPB for plants without *E. villosus* and suggested that *E. villosus* larvae did not preferentially feed on one type of seed. Benefield et al. [7] reported PB/NPB ratios of 3.9 to 10.1 for plants without *E. villosus* damage. It is not clear why *C. solstitialis* produced relatively more NPB seed to PB seed in our study populations. When all capitula over a season are combined, we estimated the efficiency of seed destruction by larval feeding from the ratio of percent total seed loss to the percentage of capitula with larvae. In our study, direct seed destruction due to larval feeding in individual capitula ranged from 62 to 95% depending on insect stage, study site, and year. However, the presence of larvae was proportionally higher in the larger capitula (>8 mm) which resulted in a disproportionately higher amount of total seed loss than would occur if the proportional rate of larval presence was the same across all size categories. As a result, the efficiency of larval feeding was over 93% for all sites and years. Interestingly, *E. villosus* showed no preference for large or small plants. 

The impact of a seed feeder on the population of its host plant needs to be examined in two ways. One is to document the direct loss of seed when the seed feeder is present. This is the focus of the study reported here. The other question is how this reduction in seed affects the population recruitment and dynamics of the plant population. This question is examined through research directed at understanding the population ecology of the host plant. It has been reported that *C. solstitialis* is seed limited [37] so seed loss due to *E. villosus* may have some impact on plant density. Swope and Satterthwaite [20] used a multi-trophic simulation model to examine the impact of *E. villosus* on *C. solstitialis* and how this impact may be affected by a generalist predatory mite which can reduce the population growth rate of the weevil. Their model results suggested that *C. solstitialis* populations would decline significantly (plant abundance was reduced 70–88% in some locations) when the weevil was allowed to increase without its natural enemy but when the mite was present, reductions in plant abundance fell in the range of 25–40%.

For *E. villosus* to cause a decline in plant abundance, it is critical that the weevil achieve high population levels where seed destruction is sufficient to result in a reduction in plant recruitment. For the four year and site combinations in our study, the total seed loss over the season was less than 47% due to the low number of capitula with larvae (range 27–49%). These observations were taken only a few years following the initial release of *E. villosus* into California, however, later studies have not shown substantial increases in intensity of attack. Woodley et al. [24] reported *E. villosus* larvae infesting 34.2% and 29.9% of capitula at a two-year field study in Washington State. Connett et al. [21] reported 32.45% of capitula with larvae for a two-year study in Idaho. Pitcairn et al. [19] surveyed the occurrence and the intensity of attack of *E. villosus* (as measured by proportion of capitula with punctures) on *C. solstitialis* throughout California in 2001 and 2002. *Eustenopus villosus* was found at 80% of the survey locations and the intensity of attack ranged from 0% to 93% with the highest values found in the mountains of northern California and along the foothills of the Sierra Nevada Mountains. Another field survey now, 30 years after *E. villosus* invaded the rangeland, may be beneficial. 

## Figures and Tables

**Table 1:** List of insect species introduced as biological control organisms on *Centaurea solstitialis* in the USA.

Species	Family	1st Release	Result
*Urophora jaculata* Rondani	Diptera: Tephritidae	1969	Failed to Establish
*Urophora sirunaseva* (Hering)	Diptera: Tephritidae	1984	Established
*Bangasternus orientalis* (Capiomont)	Coleoptera: Curculionidae	1985	Established
*Chaetorellia australis* Hering	Diptera: Tephritidae	1988	Established
*Eustenopus villosus* (Boheman)	Coleoptera: Curculionidae	1990	Established
*Larinus curtus* Hochhut	Coleoptera: Curculionidae	1992	Established

**Table 2:** Density and size (capitula/plant) of *Centaurea solstitialis* plants estimated from quadrat counts at two field sites for 1993–1995. For Nevada 1993, only density of plants was estimated so capitula per plant was obtained from plants sampled along transects and capitula per square meter was calculated by multiplying plants m^−2^ by capitula per plant. Values reported are mean (±SE) except where noted.

Site	Plants m^−2^	Capitula m^−2^	Capitula Plant^−1^
Nevada 1993	71.8 (62.1–85.2) *	631.8	8.8 (±0.9)
Nevada 1994	166.0 (±17.0)	377.2 (±48.3)	2.4 (±0.3)
Napa 1994	202.0 (±28.9)	289.2 (±34.3)	1.7 (±0.2)
Napa 1995	208.8 (±22.0)	357.2 (±35.3)	1.8 (±0.1)

* 95% confidence interval.

**Table 3:** Results of germination tests of *Centaurea solstitialis* seed from two field sites for 1993–1995. PB = pappus bearing seed; NPB = non-pappus bearing seed; N = total seed tested; Not Viable = proportion of total seed determined upon dissection to be empty or partially filled. Viable seed is the total seed tested minus the number of seed not viable.

Site	Type	N	Not Viable	Viable Seed		PB/NPB Ratio
				Did Not Germ	Germinated	
Nevada 1993	PB	14,400	5.1%	8.6%	91.4%	
	NPB	4165	5.1%	3.9%	96.1%	3.46
Nevada 1994	PB	2862	4.2%	5.2%	94.8%	
	NPB	758	4.6%	5.0%	95.0%	3.78
Napa 1994	PB	7395	4.4%	12.3%	87.7%	
	NPB	3047	2.9%	3.6%	96.4%	2.43
Napa 1995	PB	5993	5.6%	6.3%	93.7%	
	NPB	2707	5.7%	8.9%	91.1%	2.21
Napa 1995	PB	4032	7.8%	13.0%	87.0%	
control	NPB	1926	5.5%	8.6%	91.4%	2.09

**Table 4:** Capitula production and flower bud mortality from adult *Eustenopus villosus* feeding. N = number of plants. Success rate is the proportion of flower buds that become mature capitula.

Site	N	Dead Buds	Capitula	Total Buds	Success Rate
Nevada 1993	160	2500	1405	3905	36.0%
Nevada 1994	149	768	332	1100	30.2%
Napa 1994	157	189	336	525	64.0%
Napa 1995	154	620	388	1008	38.5%
Napa 1995 control	156	34	288	322	89.4%

**Table 5:** Details of *Centaurea solstitialis* plants sampled along transects and levels of attack by *Eustenopus villosus*. N = number of plants sampled; Capitula with Punctures = percentage of the seasonal total of capitula with oviposition or feeding punctures; Capitula with Larval Damage = percentage of capitula with feeding damage from *E. villosus* larvae; Punctures per Capitulum = mean (±SE) number of oviposition and feeding punctures for capitula with at least on puncture.

Site	N	Capitula per	Seeds per	Capitula with	Capitula with	Punctures per
		Plant (±SE)	Plant (±SE)	Punctures	Larval Damage	Capitulum
Nevada 1993	160	8.8 (±0.9)	84.2 (±11.7)	91.7%	35.6%	1.25 (±0.01)
Nevada 1994	149	2.2 (±0.2)	6.80 (±0.9)	78.9%	27.2%	1.58 (±0.05)
Napa 1994	157	1.7 (±0.2)	26.0 (±3.2)	68.7%	49.3%	1.21 (±0.03)
Napa 1995	154	1.8 (±0.1)	23.4 (±3.0)	80.3%	46.4%	1.12 (±0.02)
Napa 1995 control	156	1.8 (±0.2)	35.8 (±5.4)	0.0%	0.0%	0

**Table 6:** Step functions used to predict viable seed output (y) from capitulum size (x) when *Eustenopus villosus* larvae were present. The data are presented in Figure 2. Linear regression predicts seed output for capitula with the following outside diameters: Nevada 1993, 3.42–10.00 mm; Nevada 1994, 3.32–9.00 mm; Napa 1994, 2.63–9.00 mm; Napa 1995, 3.16–10.00 mm. Napa 1995 control = regression of mean viable seed output against capitulum diameter for all mature capitula. Slopes followed by the same letter are not significantly different (*p* < 0.05).

Site	y = 0	Slope (±SE)	Intercept (±SE)	Upper Range
Nevada 1993	<3.42 mm	4.58 (±0.37) a	15.69 (±2.76)	y = 36.15 for values >10.0 mm
Nevada 1994	<3.32 mm	2.17 (±0.08) b	7.20 (±0.48)	y = 8.72 for values >9.0 mm
Napa 1994	<2.63 mm	5.93 (±0.66) a	15.58 (±4.12)	y = 41.80 for values >9.0 mm
Napa 1995	<3.16 mm	4.72 (±0.38) a	15.12 (±2.81)	y = 32.80 for values >10.0 mm
Napa 1995 control		5.59 (±0.51) a	20.87 (±3.44)	

**Table 7:** Observed (with *Eustenopus villosus*) and predicted (without *E. villosus*) seed production and percent seed loss for *Centaurea solstitialis* capitula where *E. villosus* larvae completed larval development and where larvae died immature. N = number of capitula.

Site	Stage	N	Viable Seed		% Loss
			Predicted	Observed	
Nevada 1993	Pupa, Adult	201	3418	347	89.8
	Died as larva	319	5320	1265	76.2
Nevada 1994	Pupa, Adult	32	218	11	95.0
	Died as larva	56	372	53	85.8
Napa 1994	Pupa, Adult	111	3088	647	79.0
	Died as larva	55	1517	297	80.4
Napa 1995	Pupa, Adult	146	279	1496	82.2
	Died as larva	34	601	139	76.9

**Table 8:** Observed (with *Eustenopus villosus*) and predicted (without *E. villosus*) seed production and percent seed loss for transect plants at study sites from 1993 to 1995. N = number of plants sampled. Efficiency is the ratio of % seed loss to % capitula with larval damage.

Site	N	Total Seed Production		% Loss to Insects	% Capitula	Efficiency
		Observed	Predicted		with Larval Damage	
Nevada 1993	160	13,453	20,579	34.6	35.6	0.97
Nevada 1994	149	1027	1553	33.9	27.2	1.25
Napa 1994	157	4155	7816	46.8	49.3	0.95
Napa 1995	154	3623	6380	43.2	46.4	0.93

## Data Availability

The data presented in this study are available on request from the corresponding author.
